# WRKYng together: Coordination between kinase cascades and transcription factors contributes to immunity in rice

**DOI:** 10.1093/plcell/koad074

**Published:** 2023-03-13

**Authors:** Bradley Laflamme

**Affiliations:** Assistant Features Editor, The Plant Cell, American Society of Plant Biologists, USA; Department of Molecular Genetics, University of Toronto, Toronto, ON, Canada

As an early barrier against many pathogens, plants have evolved transmembrane receptors which perceive conserved motifs (or “patterns”) in microbial populations at large—things like bacterial flagellin or fungal chitin. These receptors can then activate a suite of downstream immune outputs, often collectively referred to as pattern-triggered immunity (PTI). What is particularly astonishing about PTI is the sheer variety of immune outputs and components it can encompass: calcium influx, MAP and receptor-like kinase cascades, production of reactive oxygen species, phytohormone signaling pathways, transcriptional rewiring, cell wall reinforcement, and deployment of antimicrobial compounds are just *some* of its possible outputs (Bigeard et al. 2015). The interconnectedness of all these processes is clear enough; but the precise ordering and nature of these different processes is still frequently being relitigated by the field, particularly as we see more and more examples of variation within and across plant species when it comes to these core components of PTI.

In this issue of *The Plant Cell*, **Shuai Wang and colleagues (Wang et al. 2023)** draw functional connections between MAP kinase family members, WRKY transcription factors (TFs), and auxin signaling in the rice (*Oryza sativa*) immune response. Both MAP kinases and WRKY TFs are known to be of paramount importance to plant immune pathways (Bigeard et al. 2015), though many members of these protein families have remained functionally enigmatic. Here, the authors show that WRKY31, a TF which contributes to rice resistance against *Magnaporthe oryzae* (Zhang et al. 2008), forms a functional module with a MAP kinase cascade in the rice immune system. Using several approaches (yeast-2-hybrid, protein pull-downs, and in planta bimolecular fluorescence complementation [BiFC] and co-immunoprecipitation), the authors found that WRKY31 interacts with the MAP kinase kinase MKK10-2 and several MAP kinases, including MPK3. Furthermore, these proteins formed a ternary WRKY31–MPK3–MKK10-2 complex, with in vitro and in planta assays showing that the MKK10-2/MPK3 cascade can phosphorylate and thereby increase activity of WRKY31. Knocking out MKK10-2 or WRKY31 led to increased susceptibility to the pathogen (see Figure), while overexpressing MKK10-2 improved defense only when WRKY31 was present, indicating that WRKY31 likely functions downstream of MKK10-2.

**Figure.**
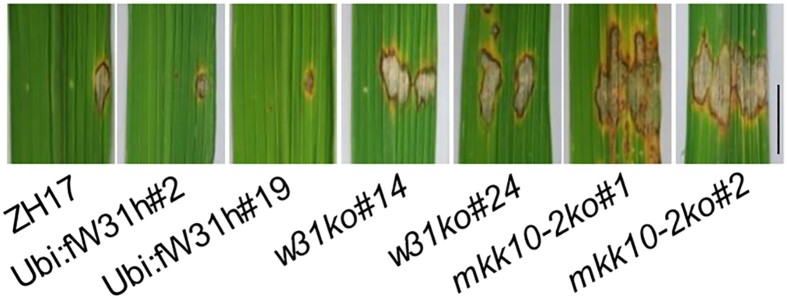
Susceptibility to *M. oryzae* is influenced by the presence of WRKY31 and MKK10-2–MAPK. From left to right: *M. oryzae* infection symptoms are shown across a range of plant genotypes: 2 WRKY31 overexpression lines (Ubi:fW31h) show lower lesion development in comparison to wild-type ZH17 plants, while 2 WRKY31 (*w31ko*) and MKK10-2 (*mkk10-2*) knockout lines show dramatic increases in lesion development. Reprinted from Wang *et al*. (2023), Supplemental Figure 5A.

Interestingly, a phosphomimic mutant allele of WRKY31 accumulated to higher levels than the wild-type allele. This led Wang et al. to investigate whether ubiquitination was controlling the stability of WRKY31. Indeed, they found that WRKY31 interacted with REIW1 (RING-finger E3 ubiquitin ligase interacting with WRKY1) in yeast, pull-downs, and in planta using BiFC. The phosphomimic mutant also interacted with REIW1, though the capacity of REIW1 to degrade this mutant is lower than the wild type. Thus, activation of WRKY31 via phosphorylation likely improves its stability and improves the quality of an immune output during infection.

The group also characterized the WRKY–MAPK–MKK module in terms of how it affects rice physiology and phytohormone accumulation. MKK10-2 overexpression in the etiolated hypocotyls of rice negatively affected the biosynthetic gene expression for, transport of, and accumulation of indole-3-acetic acid, the most common auxin hormone. In contrast, transcripts involved in the biosynthesis of jasmonic and salicylic acid, 2 other phytohormones which can antagonize auxin (Bigeard et al. 2015), were upregulated coincident with MKK10-2 overexpression. The cumulative effect of these changes was a classic “growth versus defense” trade-off: MKK10-2 activation improved resistance via its modulation of phytohormone signaling, but at the cost of plant growth.

With an investigation spanning protein–protein interactions, genetics, phytohormone analysis, biochemistry, pathology, and more, Wang and colleagues offer a compelling picture of how this module plays a significant and multifaceted role in rice PTI. Nonetheless, *M. oryzae* remains one of the most agriculturally destructive pathogens of rice worldwide, suggesting that this pathogen may have strategies for dealing with wild-type levels of WRKY31–MKK10-2 activity. A recent study of *M. oryzae* suggests that the pathogen has an expansive repertoire of virulence “effector” proteins (Yan et al. 2023), and such effectors frequently target PTI components (Bigeard et al. 2015)—MAPKs and TFs included. Thus, it will be interesting to see whether any *M. oryzae* effectors target and impair this WRKY–MAP kinase module during infection, as it may help to further decode how this nasty pathogen manages to stay ahead of PTI.
